# Impact of Preoperative Immunonutritional Support in Patients Undergoing Elective Thoracic Surgery

**DOI:** 10.31662/jmaj.2021-0095

**Published:** 2021-09-21

**Authors:** Fumihiro Shoji, Yuka Kozuma, Gouji Toyokawa, Koji Yamazaki, Sadanori Takeo

**Affiliations:** 1 Department of Thoracic Surgery, Clinical Research Institute, National Hospital Organization, Kyushu Medical Center, Fukuoka, Japan

**Keywords:** preoperative supplementation, elective thoracic surgery, body mass index, prognostic nutritional index, geriatric nutritional risk index

## Abstract

**Introduction:**

Several immunonutritional supplements have recently been developed. However, improvements in preoperative immunonutritional conditions using these supplements have not been analyzed in patients undergoing thoracic surgery.

**Methods:**

This prospective, single-arm, single-institution pilot study involved patients planning to undergo thoracic surgery. Forty adults with a poor preoperative immunonutritional status were enrolled. The patients freely selected one of three oral immunonutritional supplements (IMPACT^Ⓡ^, MEIN^Ⓡ^, or Abound^Ⓡ^) and started taking it on an outpatient basis from 7 to 14 days before thoracic surgery. The primary endpoint was the rate of improvement in three immunonutritional parameters on the hospitalization day: body mass index (BMI), prognostic nutritional index (PNI), and geriatric nutritional risk index (GNRI). These improvement rates were compared with those of a matched historical control group.

**Results:**

The PNI and GNRI improvement rates were significantly higher in the immunonutritional support group than in the control group (PNI: 103.1% ± 0.6% vs. 98.9% ± 1.3%, p = 0.0391; GNRI: 101.7% ± 0.8% vs. 99.3% ± 0.8%, p = 0.0266), although there was no significant difference in the BMI improvement rate (101.0% ± 0.6% vs. 100.2% ± 0.7%, p = 0.3626). The PNI and GNRI improvement rates were significantly higher in the IMPACT^Ⓡ^ support group than in the control group (PNI: 104.5% ± 2.4% vs. 98.9% ± 1.3%, p = 0.0212; GNRI: 101.6% ± 1.1% vs. 99.3% ± 0.8%, p = 0.0415).

**Conclusions:**

The present study revealed that short-term preoperative immunonutritional support can actually improve immunonutritional parameters immediately before surgery. In particular, preoperative immunonutritional support using IMPACT^Ⓡ^ supplementation might be the most promising agent in patients with a poor immunonutritional condition undergoing elective thoracic surgery.

**Trial registration:**

University Hospital Medical Information Network 000035851

## Introduction

Nutritional risk assessment, the impact of malnutrition, and strategies for nutritional support have been studied mainly in digestive surgery. The European Society for Parenteral and Enteral Nutrition (ESPEN) and the American Society for Parenteral and Enteral Nutrition (ASPEN) recommend preoperative nutritional risk assessment and consideration of immunonutritional supplementation for high-risk surgical patients ^(1), (2)^. The ESPEN guidelines also recommend preoperative administration of an immune-modulating diet enriched with arginine, nucleotides, and ω-3 fatty acids (O3FA) to patients with malnutrition ^(1)^. In fact, several recently reported trials have suggested that preoperative administration of an immunonutritional supplement is necessary in improving clinical outcomes in patients undergoing surgical resection of cancer, mainly digestive cancer ^(3), (4)^.

Several objective immunonutritional parameters have been developed ^(5)^. A patient’s body weight reflects his or her metabolism. Serum albumin is the simplest and most valuable parameter for assessing the nutritional status, and lymphocytes play a fundamentally important role in host immune responses ^(6), (7), (8)^. Therefore, the body mass index (BMI) is one of the most widely used tools for assessment of patients’ nutritional condition ^(1), (2)^. The geriatric nutritional risk index (GNRI) is based on two parameters: the serum albumin concentration and the ideal body weight calculated based on BMI ^(9)^. Thus, like BMI, GNRI might reflect patients’ nutritional status. The prognostic nutritional index (PNI) is a particularly attractive immunonutritional biomarker. It is calculated from the serum albumin concentration and lymphocyte count in the peripheral blood ^(10)^. Thus, PNI is a measure of both the nutritional and immunological conditions of a patient.

Associations between elective thoracic surgery and patients’ immunonutritional condition are being investigated. Most studies of the immunonutritional condition of patients undergoing thoracic surgery have focused on the possibility of this condition as a predictive factor of perioperative complications or a prognostic factor. In fact, we previously reported that the preoperative immunonutritional status is a prognostic factor in patients with early-stage lung cancer ^(11), (12), (13)^. We have also demonstrated that the preoperative immunonutritional status is a novel preoperative predictor of postoperative comorbidities and a prognostic factor that may identify high risk among advanced-age patients with lung cancer ^(14)^.

According to our above-mentioned studies, we consider three treatment strategies for patients with lung cancer who have a poor immunonutritional condition. First, we immunonutritionally support these patients before thoracic surgery and perform radical resections. Second, we perform limited resections for these patients. Third, we avoid surgical resections for these patients and select alternative therapies such as radiotherapy, chemotherapy, and palliative care. Nevertheless, few studies have objectively evaluated immunonutritional support in patients undergoing thoracic surgery, and recommendations for preoperative assessment of these patients do not address nutritional assessment or the provision of nutritional support. Moreover, little information is available regarding the best immunonutritional supplement for patients undergoing thoracic surgery.

Therefore, it is crucial to clarify the possibility of improving the immunonutritional condition in patients with a poor immunonutritional status undergoing elective lung cancer surgery by performing preoperative immunonutritional support within a limited time. However, few studies have objectively analyzed improvements in immunonutritional parameters obtained by only short-term preoperative immunonutritional support. We, therefore, examined whether the preoperative immunonutritional condition of patients with a poor immunonutritional status undergoing elective thoracic surgery is actually improved by immunonutritional supplementation.

We prospectively analyzed the improvements in preoperative immunonutritional parameters (BMI, GNRI, and PNI) in patients undergoing elective thoracic surgery using short-term preoperative immunonutritional support using three well-recognized immunonutritional supplements.

## Materials and Methods

### Study design

This was a pilot study with a prospective, single-arm design conducted at a single institution (Department of Thoracic Surgery, Clinical Research Institute, National Hospital Organization Kyushu Medical Center). All participants were candidates for elective thoracic surgery from January 2019 to September 2019. The patients were considered eligible if they met one or more of the following three inclusion criteria based on previously described poor immunonutritional criteria ^(1), (2), (15)^: preoperative blood lymphocyte count < 1,600/μl, preoperative serum albumin level < 3.5 g/dl, and <90% of standard body weight. The exclusion criteria were active preoperative viral or bacterial infection or other inflammatory conditions as evaluated by the patients’ symptoms, hematological and urinary tests, and imaging results (e.g., X-ray or computed tomography); treatment with adrenocorticosteroids; and a history of recent immunosuppressive or immunological diseases.

### Approval by an institutional review board

The study protocol was reviewed and approved by the institutional review board (IRB) of National Hospital Organization Kyushu Medical Center (approved IRB number: 18A242), and all participants provided written informed consent. The study was conducted in accordance with the Declaration of Helsinki. This study was registered in the Clinical Trial Registry (University Hospital Medical Information Network: UMIN 000035851).

### Protocol

**Figure 1.**
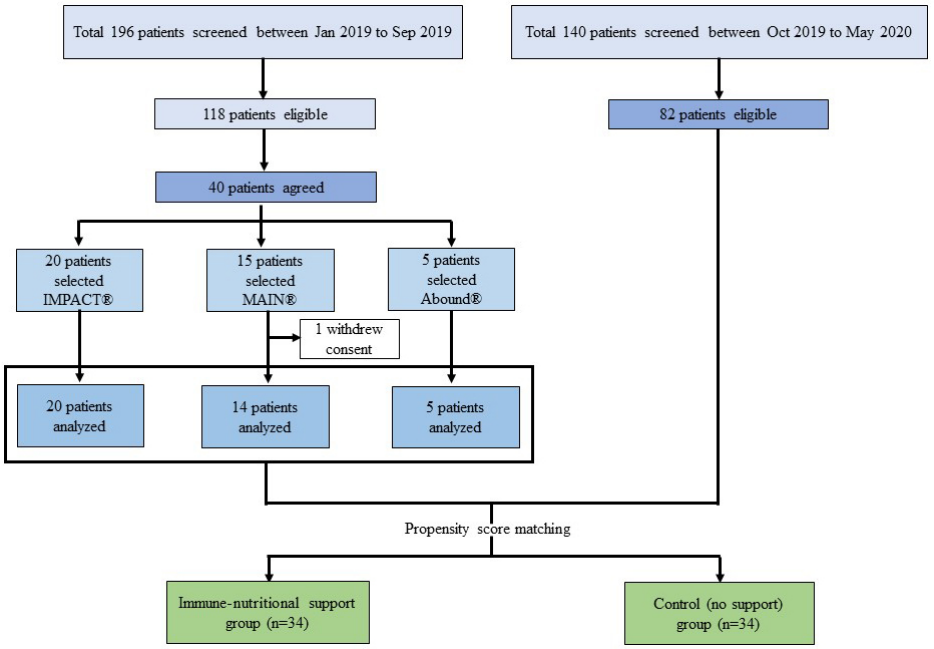
Flow chart.

A flow chart is presented in Figure 1. In total, 40 patients were finally enrolled. The detailed inclusion criteria of these patients are presented in Supplementary Figure 1. We used the following three new oral immunonutritional supplements: (1) Some patients received oral supplementation (440 kcal/day) containing arginine, O3FA, and dietary nucleotides (oral IMPACT^Ⓡ^; Nestle Health Science Co., Ltd., Kobe, Japan) for 7 days before surgery, as previously described ^(4)^. A daily total of 600 mL of oral IMPACT^Ⓡ^ contains 9.6 g of arginine, 1.76 g of O3FA, and 0.96 g of dietary nucleotides. (2) Some patients received oral supplementation (600 kcal/day) containing whey hydrolyzed protein, amino acids, 21% medium-chain triglycerides, enriched O3FA, ω-6 fatty acids (O6FA), and vitamins C and E (oral MEIN^Ⓡ^; Meiji Dairies Co., Tokyo, Japan) for 5 days before surgery, as previously described ^(16), (17)^. A daily total of 600 mL of oral MEIN^Ⓡ^ contains 30.0 g of whey hydrolyzed protein; 0.90 g of arginine; 6.54 g of glutamine; 2.94 g of aspartic acid; 2.88 g of leucine; O3FA containing 4% alpha linoleic acid, 1.2% eicosapentaenoic acid, and 0.8% docosahexaenoic acid; and O6FA containing 12% linoleic acid. (3) Some patients received oral supplementation (158 kcal/day) containing beta-hydroxy-beta-methyl-butyric-acid (HMB), l-glutamine, and l-arginine (oral Abound^Ⓡ^; Abbott Japan Co., Tokyo, Japan) for 14 days before surgery, as previously described ^(18)^. A daily total of 600 mL of oral Abound^Ⓡ^ contains 2.4 g of HMB, 14 g of l-arginine, and 14 g of l-glutamine. The patients freely selected one of these three supplements. Twenty patients (50.0%) selected IMPACT^Ⓡ^; 15 (37.5%) selected MEIN^Ⓡ^; and 5 (12.5%) selected Abound^Ⓡ^. Finally, 39 patients were analyzed because one patient withdrew consent. The control group comprised 82 eligible patients who underwent thoracic surgery from October 2019 to May 2020. After one-to-one propensity score matching, 34 patients in the immunonutritional support group were compared with 34 patients in the matched control group. The primary endpoint was the rate of improvement in the preoperative immunonutritional parameters.

### Laboratory analyses

Blood samples were obtained the day before the start of preoperative immunonutritional support (the day of the first outpatient visit in the control group) and the day before surgery.

### Preoperative calculation of immunonutritional parameters (BMI, GNRI, and PNI)

The BMI was calculated using the following formula: body weight (kg) / body height^2^ (m). The GNRI was calculated from the serum albumin level and body weight using the following formula: GNRI = 14.87 × serum albumin level (g/L) + 41.7 × preoperative weight (kg) / ideal weight (kg). The ideal body weight was calculated as follows: ideal body weight = 22 × height (m^2^) ^(9)^. The PNI was calculated based on two parameters (total lymphocyte count in peripheral blood and serum albumin level) using the following formula: 10 × serum albumin level (g/dL) + 0.005 × total peripheral blood lymphocyte count (per mm^3^) ^(10)^.

### Statistical analysis

Categorical variables were analyzed using Fisher’s exact test. Continuous variables are expressed as mean and standard error, and their means were compared using the chi-squared test. Categorical variables were analyzed using the chi-squared test. Propensity score matching was performed to reduce the potential for bias introduced by non-random comparison of the immunonutritional support group and control group. To calculate the propensity scores, we fitted a logistic regression model using the following five variables: age, sex, smoking status, preoperative comorbidities, and preoperative diagnosis. To find matched patients in the groups, one-to-one greedy matching was carried out using a caliper width 0.20 of the pooled standard deviation of the logit of the propensity score. A p value < 0.05 was considered significant. All statistical analyses were performed using JMP software, version 14.0 (SAS Institute, Inc., Cary, NC, USA).

## Results

### Details of 39 patients receiving immunonutritional support

Table 1 shows the patients’ clinicopathological profiles. The study group comprised 19 women and 20 men with a mean age at the time of surgery of 72 years (range, 21-89 years). Thirty-five patients (89.7%) had a European Cooperative Oncology Group performance status (ECOG-PS) of 0, and 4 (10.3%) had ECOG-PS of 1. Nineteen patients (48.7%) had never smoked, and the remaining 20 were current or former smokers. Their preoperative diagnoses were primary lung cancer in 26 patients (67.5%), metastatic lung tumors in 9 (22.5%), and other benign diseases in 4 (10.0%). Of the 39 patients, 28 (71.8%) patients had a history, 3 (33.3%) had hypertension, 8 (20.5%) had type 2 diabetes mellitus, 8 (20.5%) had other organ malignancies, and 6 (15.4%) had cerebral infarction. A total of 24 patients (61.5%) took medications to treat these diseases.

**Table 1.** Patients’ Baseline Characteristics.

Items		No. (%) or Median (Range)
Gender	Male	20 (52.5)
	Female	19 (47.5)
Age		72 (21-89)
ECOG-PS	0	35 (89.7)
	1	4 (10.3)
Body balance	Body height (cm)	158 (140-176)
	Body weight (kg)	56.6 (37.0-86.0)
Preoperative diagnosis	Lung cancer	26 (67.5)
	Metastatic lung tumors	9 (22.5)
	Others	4 (10.0)
Smoking status	Non	19 (48.7)
	Current	3 (7.7)
	Former	17 (43.6)
Comorbidities	Hypertension	13 (33.3)
	Type 2 diabetes mellitus	8 (20.5)
	Other malignancies	8 (20.5)
	Cerebral infarction	6 (15.4)

ECOG-PS: European Cooperative Oncology Group performance status

### Changes in body weight and hematological data after receiving immunonutritional support

Table 2 shows changes in the body weight and hematological data of all 39 patients after they received immunonutritional support. The mean body weight was 58.9 ± 1.9 kg at baseline and 59.4 ± 1.9 kg immediately before surgery. The mean difference in the body weight after immunonutritional support was 0.4 (95% confidence interval [CI], −0.2 to 1.0 kg). The mean peripheral blood lymphocyte count was 1282/μL ± 57/μL at baseline and 1384/μL ± 76/μL immediately before surgery. The mean difference in the peripheral blood lymphocyte count after immunonutritional support was 102.0/μL (95% CI, −15.4/μL to 219.5/μL). The mean serum albumin concentration was 4.1 ± 0.1 g/dL at baseline and 4.2 ± 0.1 g/dL immediately before surgery. The mean difference in the serum albumin concentration after immunonutritional support was 0.1 (95% CI, 0.0-0.2 g/dL).

**Table 2.** Changes in Body Weight and Hematological Data after Immunonutritional Support.

Items	Baseline	After support	Mean difference (95% CI)
Body weight (kg)	58.9 ± 1.9	59.4 ± 1.9	0.4 (−0.2 to 1.0)
Complete blood cell counts			
White blood cell count (/μL)	5.582 ± 291	5,726 ± 257	143.6 (−216.4 to 503.6)
Neutrophils (/μL)	3,724 ± 233	3,776 ± 221	51.8 (−281.3 to 384.8)
Lymphocytes (/μL)	1,282 ± 57	1,384 ± 76	102.0 (−15.4 to 219.5)
Monocytes (/μL)	324 ± 20	355 ± 21	31.5 (−1.1 to 64.1)
Eosinophils (/μL)	231 ± 60	172 ± 28	−59.2 (−169.1 to 50.8)
Basophils (/μL)	36 ± 3	37 ± 4	1.7 (−3.4 to 6.9)
Hemoglobin (g/dL)	12.6 ± 0.3	12.7 ± 0.3	0.1 (−0.2 to 0.3)
Hematocrit (%)	38.0 ± 0.8	38.1 ± 0.8	0.0 (−0.5 to 0.6)
RDW (%)	14.3 ± 0.5	14.1 ± 0.3	−0.2 (−0.7 to 0.3)
Platelet (× 10^3^/μL)	218 ± 12	217 ± 11	−0.5 (−11.5 to 10.4)
Serum chemistry			
Albumin (g/dL)	4.1 ± 0.1	4.2 ± 0.1	0.1 (0.0 to 0.2)
Total cholesterol (mg/dL)	196 ± 10	191 ± 5	−5.0 (−12.7 to 2.7)
Triglyceride (mg/dL)	140 ± 17	109 ± 7	38.4 (−0.4 to 77.3)
Blood sugar (mg/dL)	115 ± 8	105 ± 5	18.0 (1.8 to 34.2)
ALP (U/L)	244 ± 15	241 ± 13	−0.8 (−14.3 to 12.7)
LDH (U/L)	197 ± 6	209 ± 6	−10.8 (−17.8 to −3.8)
AST (U/L)	24 ± 2	25 ± 2	−0.9 (−2.7 to 0.8)
ALT (U/L)	23 ± 4	25 ± 4	−2.2 (−5.0 to 0.7)
G-GTP (U/L)	36 ± 6	40 ± 6	−5.4 (−10.5 to −0.4)
BUN (mg/dL)	17 ± 1	23 ± 2	−6.4 (−9.1 to −3.6)
Cr (mg/dL)	0.84 ± 0.05	0.82 ± 0.05	0.0 (−0.0 to 0.1)
CRP (mg/dL)	0.34 ± 0.14	0.26 ± 0.09	0.1 (−0.1 to 0.3)

Data are presented as mean ± standard error. CI: confidence interval, RDW: red cell distribution width, ALP: alkaline phosphatase, LDH: lactate dehydrogenase, AST: aspartate transaminase, ALT: alanine transaminase, G-GTP: gamma-glutamyl transpeptidase, BUN: blood urea nitrogen, Cr: creatinine, CRP: C-reactive protein

### Improvements in immunonutritional parameters after receiving immunonutritional support

Table 3 shows improvements in the three immunonutritional parameters after the patients received immunonutritional support. After immunonutritional support, the BMI changed from 23.0 ± 0.5 to 22.5 ± 0.8 kg/m^2^. The mean difference in the BMI after immunonutritional support was −0.2 kg/m^2^ (95% CI, −0.4 to 0.1 kg/m^2^). GNRI changed from 104.1 ± 1.2 to 104.5 ± 1.9. The mean difference in GNRI after immunonutritional support was 1.8 (95% CI, 0.3-3.2). PNI changed from 40.8 ± 0.7 to 41.7 ± 0.7. The mean difference in PNI after immunonutritional support was 1.5 (95% CI, 0.2-2.7).

**Table 3.** Improvement Rates in Immunonutritional Parameters after Immunonutritional Support.

Items	Baseline	After support	Mean difference (95% CI)
Body mass index	23.0 ± 0.5	22.5 ± 0.8	−0.2 (−0.4 to 0.1)
Geriatric nutritional risk index	104.1 ± 1.2	104.5 ± 1.9	1.8 (0.3 to 3.2)
Prognostic nutritional index	40.8 ± 0.7	41.7 ± 0.7	1.5 (0.2 to 2.7)

Data are presented as mean ± standard error. CI: confidence interval

### Comparison of immunonutritional parameters between the control group and the immunonutritional support group

**Figure 2.**
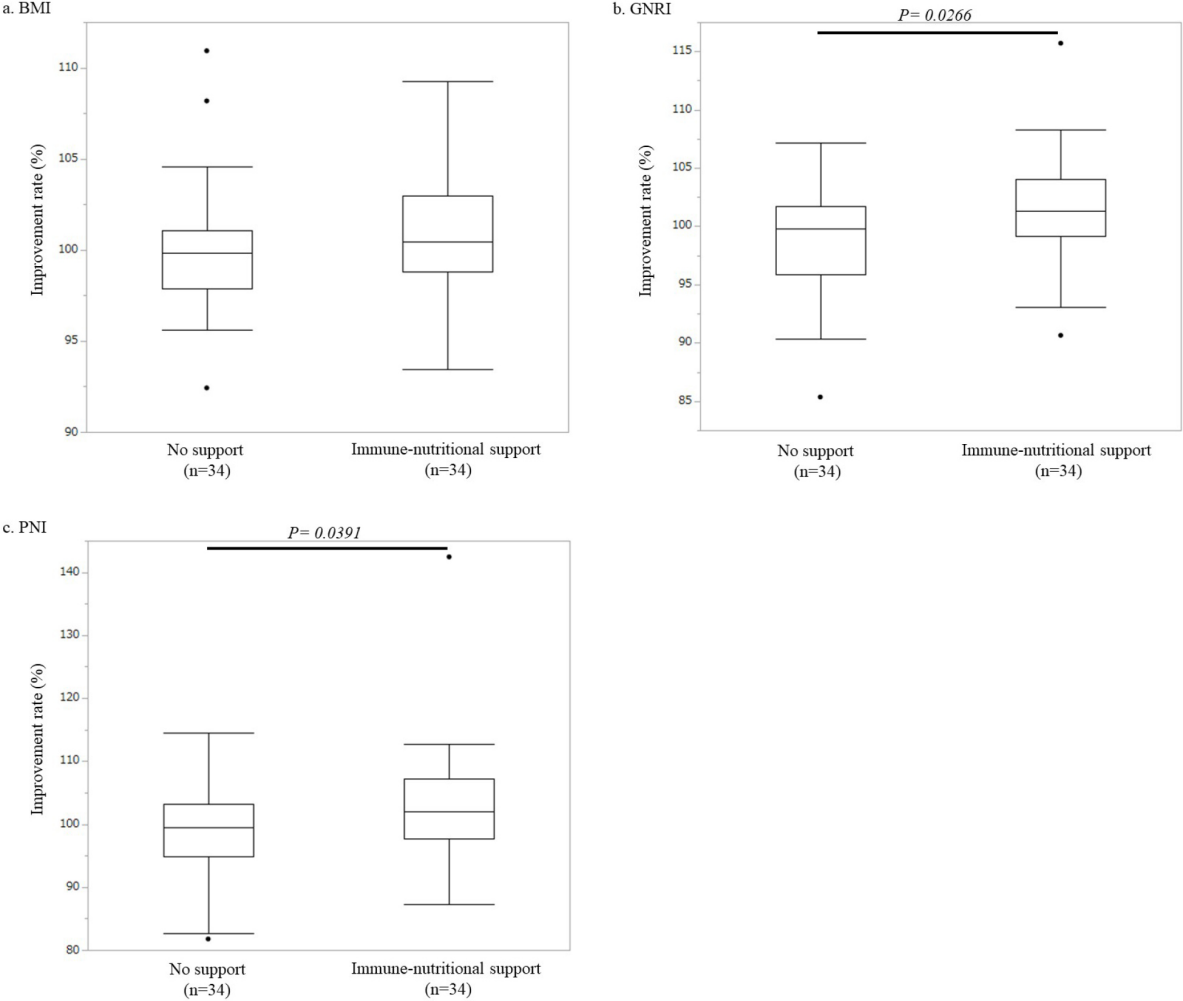
Box plot shows the rate of improvement in the control group (n = 34) and the immunonutritional support group (n = 34). (a) BMI: There was no significant difference between the two groups. (b) GNRI: The rate of improvement was significantly higher in the immunonutritional support group than in the control group (p = 0.0124). (c) PNI: The rate of improvement was significantly higher in the immunonutritional support group than in the control group (p = 0.0379).

After adjusting for background factors by propensity score matching, clinical features such as sex, age, ECOG-PS, smoking status, preoperative comorbidities, and preoperative diagnosis were similar between the control group and the immunonutritional support group (Table 4). Although there was no significant difference in the BMI improvement rate between the immunonutritional support group and the control group (101.0% ± 0.6% vs. 100.2% ± 0.7%, p = 0.3626) (Figure 2a), GNRI and PNI improvement rates were significantly higher in the immunonutritional support group than in the control group (GNRI: 101.7% ± 0.8% vs. 99.3% ± 0.8%, p = 0.0266; PNI: 103.1% ± 0.6% vs. 98.9% ± 1.3%, p = 0.0391) (Figure 2b and c).

**Table 4.** Patients’ Baseline Characteristics in Both Groups after Propensity Score Matching.

Items	Control group (n = 34)	Immunonutritional support group (n = 34)	*P value*
Gender			*0.3306*
Male	14	18
Female	20	16
Age, median (range)	74 (49-87)	74 (46-89)	*0.7540*
ECOG-PS			*0.7337*
0	28	30	
1	6	4	
Smoking status			*0.7854*
Non	18	16	
Former	15	16	
Current	1	2	
Preoperative comorbidities			*0.7794*
No	8	9	
Yes	26	25	
Preoperative diagnosis			*0.9525*
Lung cancer	25	24	
Metastatic lung tumors	6	7	
Others	3	3	
*Immunonutritional supplements*			*-*
IMPACT^Ⓡ^	-	19	
MEIN^Ⓡ^	-	12	
Abound^Ⓡ^	-	3	

ECOG-PS: European Cooperative Oncology Group performance status

### Comparison of immunonutritional parameters according to supplement type

**Figure 3.**
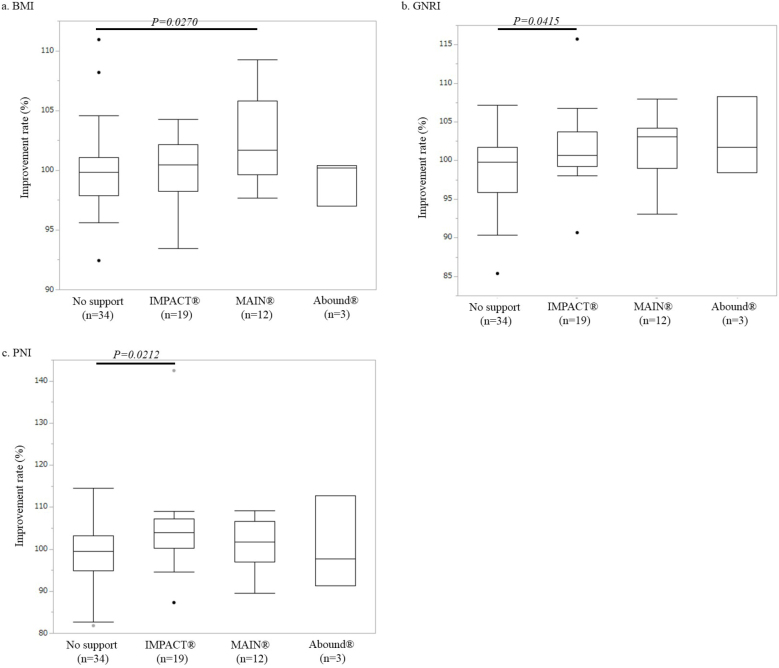
Box plot shows the improvement rate in the control group (n = 34) and each immunonutritional support group [the IMPACT^Ⓡ^ support group (n = 19), the MEIN^Ⓡ^ support group (n = 12), and the Abound^Ⓡ^ support group (n = 3)]. (a) BMI: The improvement rate was significantly higher in the MEIN^Ⓡ^ support group than in the control group (p = 0.0270). (b) GNRI: The improvement rate was significantly higher in the IMPACT^Ⓡ^ support group than in the control group (p = 0.0415). (c) PNI: The improvement rate was significantly higher in the IMPACT^Ⓡ^ support group than in the control group (p = 0.0212).

Figure 3 illustrates the immunonutritional parameter improvement rates in each immunonutritional group. The GNRI and PNI improvement rates were significantly higher in the IMPACT^Ⓡ^ support group than in the control group (GNRI: 101.6% ± 1.1% vs. 99.3% ± 0.8%, p = 0.0415; PNI: 104.5% ± 2.4% vs. 98.9% ± 1.3%, p = 0.0212). However, there was no significant difference in the BMI improvement rate compared with that in the control group (100.0% ± 0.7% vs. 100.2% ± 0.7%, p = 0.9172). The BMI improvement rate was significantly higher in the MEIN^Ⓡ^ support group than in the control group (102.6% ± 1.0% vs. 100.2% ± 0.7%, p = 0.0270). However, there was no significant difference in the GNRI or PNI improvement rate compared with that in the control group (GNRI: 101.5% ± 1.2% vs. 99.3% ± 0.8%, p = 0.0764; PNI: 100.9% ± 1.7% vs. 98.9% ± 1.3%, p = 0.3705). There were no significant differences in any of the three immunonutritional parameters between the Abound^Ⓡ^ support group and the control group (BMI: 99.2% ± 1.1% vs. 100.2% ± 0.7%, p = 0.7070; GNRI: 102.8% ± 2.9% vs. 99.3% ± 0.8%, p = 0.1314; PNI: 100.5% ± 6.4% vs. 98.9% ± 1.3%, p = 0.6935).

## Discussion

Many studies have presented patients’ clinical outcomes without objective confirmation of whether the immunonutritional parameters immediately before surgery were truly improved by preoperative immunonutritional support. Thus, our data are meaningful for clarification of the objective evaluation of the effects of preoperative immunonutritional support. Our study also demonstrated the impact of immunonutritional support using preoperative immunonutritional supplements in patients with a poor immunonutritional condition undergoing elective thoracic surgery.

The present pilot study has two novel findings. First, it demonstrated that preoperative short-term immunonutritional support can actually improve immunonutritional parameters, including PNI and GNRI, immediately before surgery. The preoperative time spent on immunonutritional support is limited even in patients undergoing elective thoracic surgery, especially patients with lung cancer or metastatic lung tumors. Spending more time on improving the preoperative immunonutritional condition may increase the risk of progression of these malignancies. Thus, we doubted whether the immunonutritional condition could be improved by only preoperative immunonutritional support for 7 to 14 days before surgery. We found that both GNRI and PNI, which are calculated using the serum albumin level, peripheral blood lymphocyte count, and body weight, were significantly improved by only short-term immunonutritional support. The second novel finding is that IMPACT^Ⓡ^ seems to be a promising immunonutritional supplement among the three immunonutritional support agents evaluated. Preoperative immunonutritional support using IMPACT^Ⓡ^ might be beneficial for patients with a poor immunonutritional condition undergoing elective thoracic surgery, although its usefulness will need to be verified by future randomized studies.

IMPACT^Ⓡ^ is an immunonutritional supplement mainly composed of arginine, O3FA, and nucleotides and has been widely used as a perioperative immunonutritional supplement, mainly in patients undergoing digestive surgery. A meta-analysis ^(4)^ indicated that the risk of postoperative complications and mortality after surgery for gastrointestinal cancers was significantly reduced in patients who had received preoperative immunonutritional support using IMPACT^Ⓡ^.

As mentioned above, IMPACT^Ⓡ^ has shown promising results mainly in patients undergoing digestive surgery. Nevertheless, a few studies have shown not only actual improvement data of immunonutritional parameters by preoperative immunonutritional support but also the usefulness of these agents for thoracic surgery patients. Kaya et al. ^(19)^ reported that preoperative immunonutritional support using a regimen enriched with arginine, O3FA, and nucleotides for 10 days reduced postoperative complications in thoracic surgery patients. They also showed that the reduction rate of the postoperative serum albumin concentration on postoperative day 3 compared with that at baseline (before immunonutritional support) was decreased by preoperative immunonutritional support. However, the study excluded patients with malnutrition who truly needed immunonutritional support, and it lacked immunonutritional data including BMI, PNI, and GNRI at hospitalization (immediately before thoracic surgery). Therefore, whether preoperative immunonutritional support could improve the preoperative immunonutritional condition immediately before thoracic surgery was unclear.

International guidelines such as those established by ESPEN and ASPEN include weight loss of >10% as part of the definition of a malnutritional condition ^(1), (2)^. In addition, we adopted the criteria of a low blood lymphocyte count (1,600/mm^3^) and albumin concentration level (3.5 g/dl) as defined by De Ulíbarri et al. ^(15)^. Based on our inclusion criteria, most enrolled patients undergoing elective thoracic surgery (36 of 39 patients, 92.3%) had “hypo-lymphocytopenia” rather than “malnutrition” such as body weight loss or hypoalbuminemia (Supplementary Figure 1). Therefore, patients undergoing elective thoracic surgery seem to clearly differ from those undergoing digestive surgery. In other words, a “poor immunological condition” rather than “malnutrition” should be given more attention in patients undergoing thoracic surgery. A multicenter randomized clinical trial ^(20)^ revealed that implementation of a protocol including immunonutritional supplementation reduced complications in patients undergoing colorectal surgery compared with a protocol including classic nutritional supplementation. Thus, it may be reasonable for patients undergoing elective thoracic surgery to use not simple nutritional supplements but instead immunonutritional supplements. In the present study, only the IMPACT^Ⓡ^ supplement contributed to improvement in the PNI, which is composed of the serum albumin level and the total peripheral blood lymphocyte count. In particular, the patient as the extreme outlier, illustrated in Figure 2b, 2c, 3b and 3c, received preoperative immunonutritional support using IMPACT^Ⓡ^ supplementation. The laboratory data of this patient before preoperative immunonutritional support showed both hypo-albuminemia and a low serum lymphocyte account. Both PNI and GNRI were drastically improved by IMPACT^Ⓡ^ supplementation (improvement rate; PNI = 139.43%, post-PNI/pre-PNI = 46.12/33.07 and GNRI = 115.69%; post-GNRI/pre-GNRI = 123.43/106.69, respectively). Thus, among the three supplements we analyzed, IMPACT^Ⓡ^ seems to be promising for patients with a poor immunonutritional condition undergoing elective thoracic surgery.

Lastly, we also analyzed the actual postoperative effects of preoperative immunonutritional support, such as the postoperative time course of immunonutritional parameters including serum albumin concentration and peripheral lymphocytes count, and the occurrence rate of postoperative complications using the propensity score-matched analysis, although these results were only for reference purposes. Supplementary Figure 2 illustrates the time course of both serum albumin concentration and peripheral lymphocyte count. Preoperative immune-nutritional support tended to preserve the serum albumin concentration on postoperative day 3 (control group vs. immunonutritional support group = 3.1 ± 0.1 g/dL vs. 3.3 ± 0.1 g/dL, p = 0.0971). Moreover, the occurrence rate of postoperative complications (>grade II according to the Clavian-Dindo classification) ^(21)^ is illustrated in Supplementary Figure 3. The rates in the control group and the immune-nutritional support group were 11.76% (8 of 34 patients) and 7.35% (5 of 34 patients), respectively, which short preoperative immunonutritional support might decrease the occurrence rate of postoperative complications even in thoracic surgery patients.

Notably, this was a single-institution pilot study. Using the knowledge obtained from the present study, a multicenter large-scale prospective randomized study should be conducted to evaluate whether preoperative immunonutritional support using IMPACT^Ⓡ^ supplementation can prevent postoperative complications and improve the postoperative prognosis of patients with a poor immunonutritional condition undergoing elective thoracic surgery.

### Conclusions

In conclusion, our results indicate that preoperative immunonutritional support is promising and reasonable. In particular, preoperative immunonutritional support using IMPACT^Ⓡ^ supplementation might be especially promising in patients with a poor immunonutritional condition undergoing elective thoracic surgery.

## Article Information

### Conflicts of Interest

The authors declare that they have no conflict of interest. IMPACT^Ⓡ^ was provided by Nestle Health Science Co., Ltd., Kobe, Japan; MEIN^Ⓡ^ was provided by Meiji Dairies Co., Tokyo, Japan; and Abound^Ⓡ^ was provided by Abbott Japan Co., Tokyo, Japan.

### Acknowledgement

We thank Angela Morben, DVM, ELS, from Edanz Group (https://en-author-services.edanzgroup.com/) for editing a draft of this manuscript.

### Author Contributions

The corresponding author and coauthors contributed according to the following four criteria: 1. substantial contributions to the conception or design of the work or the acquisition, analysis, or interpretation of data for the work; 2. drafting the work or revising it critically for important intellectual content; 3. final approval of the version to be published; and 4. agreement to be accountable for all aspects of the work in ensuring that questions related to the accuracy or integrity of any part of the work are appropriately investigated and resolved.

### Data Sharing Statement

All data collected for the study, including individual participant data and a data dictionary defining each field in the set, will be made available to others. Clinical trial registration: https://www.umin.ac.jp.

## Supplement

Supplementary Figure 1 Pie chart showing the enrolled reasons in the present study based on our inclusion criteria.

Supplementary Figure 2 The postoperative time course of immune-nutritional parameters including serum albumin concentration on postoperative day (POD) 1 (A) and POD 3 (B) and peripheral lymphocytes count on POD 1 (C) and POD 3 (D) divided by no-support (control) group and preoperative immunonutritional support group. Data are presented as mean ± standard error.

Supplementary Figure 3 A bar chart showing the occurrence of postoperative complications divided by the no-support (control) group and the preoperative immunonutritional support group.

## References

[1] Weimann A, Braga M, Carli F, et al. ESPEN guideline: clinical nutrition in surgery. Clin Nutr. 2017;36(3):623-50. 28385477 10.1016/j.clnu.2017.02.013

[2] McClave SA, Taylor BE, Martindale RG, et al. Guidelines for the provision and assessment of nutrition support therapy in the adult critically ill patient: Society of Critical Care Medicine (SCCM) and American Society for Parenteral and Enteral Nutrition (A.S.P.E.N.). JPEN J Parenter Enteral Nutr. 2016;40(2):159-211. 26773077 10.1177/0148607115621863

[3] Cerantola Y, Hübner M, Grass F, et al. Immunonutrition in gastrointestinal surgery. Br J Surg. 2011;98(1):37-48. 20931620 10.1002/bjs.7273

[4] Adiamah A, Skořepa P, Weimann A, et al. The impact of preoperative immune modulating nutrition on outcomes in patients undergoing surgery for gastrointestinal cancer: a systematic review and meta-analysis. Ann Surg. 2019;270(2):247-56. 30817349 10.1097/SLA.0000000000003256

[5] Shoji F. Clinical impact of preoperative immunonutritional status in patients undergoing surgical resection of lung cancer. J Thorac Dis. 2019;11(Suppl 3):S408-12. 30997233 10.21037/jtd.2018.11.118 PMC6424752

[6] Coussens LM, Werb Z. Inflammation and cancer. Nature. 2002;420(6917):860-7. 12490959 10.1038/nature01322 PMC2803035

[7] Mantovani A, Allavena P, Sica A, et al. Cancer-related inflammation. Nature. 2008;454(7203):436-44. 18650914 10.1038/nature07205

[8] Roxburgh CS, McMillan DC. Role of systemic inflammatory response in predicting survival in patients with primary operative cancer. Future Oncol. 2010;6(1):149-63. 20021215 10.2217/fon.09.136

[9] Bouillanne O, Morineau G, Dupont C, et al. Geriatric nutritional index: a new index for evaluating at-risk elderly medical patients. Am J Clin Nutr. 2005;82(4):7777-83. 10.1093/ajcn/82.4.777 16210706

[10] Onodera T, Goseki N, Kosaki G. Prognostic nutritional index in gastrointestinal surgery of malnourished cancer patients. Nihon Geka Gakkai Zasshi. 1984;85(9):1001-5. 6438478

[11] Shoji F, Morodomi Y, Akamine T, et al. Predictive impact for postoperative recurrence using the preoperative prognostic nutritional index in pathological stage I non-small cell lung cancer. Lung Cancer. 2016;98:15-21. 27393501 10.1016/j.lungcan.2016.05.010

[12] Shoji F, Haratake N, Akamine T, et al. The preoperative controlling nutritional status score predicts survival after curative surgery in patients with pathological stage I non-small cell lung cancer. Anticancer Res. 2017;37(2):741-7. 28179325 10.21873/anticanres.11372

[13] Shoji F, Matsubara T, Kozuma Y, et al. Preoperative geriatric nutritional risk index: a predictive and prognostic factor in patients with pathological stage I non-small cell lung cancer. Surg Oncol. 2017;26(4):483-88. 29113668 10.1016/j.suronc.2017.09.006

[14] Shoji F, Miura N, Matsubara T, et al. Prognostic significance of immune-nutritional parameters for surgically resected elderly lung cancer patients: a multicentre retrospective study. Interact Cardiovasc Thorac Surg. 2018;26(3):389-94. 29049803 10.1093/icvts/ivx337

[15] Ignacio de Ulíbarri J, González-Madroño A, de Villar NG, et al. CONUT: a tool for controlling nutritional status. First validation in a hospital population. Nutr Hosp. 2005;20(1):38-45. 15762418

[16] Kitagawa H, Namikawa T, Yatabe T, et al. Effects of a preoperative immune-modulating diet in patients with esophageal cancer: a prospective parallel group randomized study. Langenbecks Arch Surg. 2017;402(3):531-8. 28283752 10.1007/s00423-016-1538-5

[17] Oner OZ, Oğünç AV, Cingi A, et al. Whey feeding suppresses the measurement of oxidative stress in experimental burn injury. Surg Today. 2006;36(4):376-81. 16554996 10.1007/s00595-005-3166-5

[18] Hsieh LC, Chien SL, Huang MS, et al. Anti-inflammatory and anticatabolic effects of short-term beta-hydroxy-beta-methylbutyrate supplementation on chronic obstructive pulmonary disease patients in intensive care unit. Asia Pac J Clin Nutr. 2006;15(4):544-50. 17077073

[19] Kaya SO, Akcam TI, Ceylan KC, et al. Is preoperative protein-rich nutrition effective on postoperative outcome in non-small cell lung cancer surgery? A prospective randomized study. J Cardiothorac Surg. 2016;11(1):14. 26782276 10.1186/s13019-016-0407-1 PMC4717613

[20] Moya P, Soriano-Irigaray L, Ramirez JM, et al. Perioperative standard oral nutrition supplements versus immunonutrition in patients undergoing colorectal resection in an enhanced recovery (ERAS) protocol: a multicenter randomized clinical trial (SONVI Study). Medicine (Baltimore). 2016;95(21):e3704. 27227930 10.1097/MD.0000000000003704 PMC4902354

[21] Dindo D, Demartines N, Clavien PA. Classification of surgical complications: a new proposal with evaluation in a cohort of 6336 patients and results of a survey. Ann Surg. 2004;240(2):205-13. 15273542 10.1097/01.sla.0000133083.54934.ae PMC1360123

