# Supplementary figures and images for: Impact of Preoperative Immunonutritional Support in Patients Undergoing Elective Thoracic Surgery
Source: JMA J. 2021 Sep 21;4(4):387–96. doi: 10.31662/jmaj.2021-0095 (PMC8580703; doi:10.31662/jmaj.2021-0095)

Supplementary fig 1.

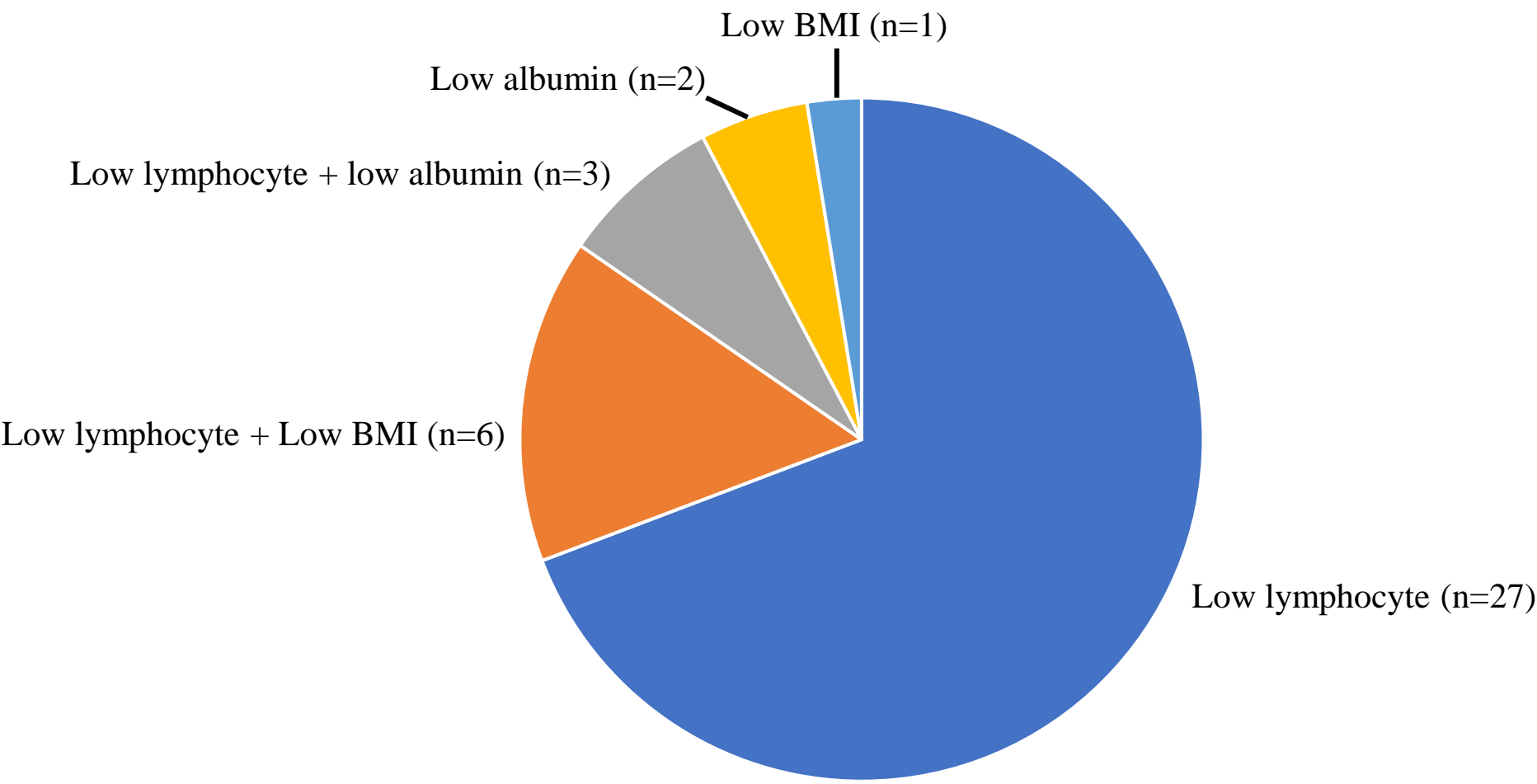

Supplement: Supplementary Figure 1 — Pie chart showing the enrolled reasons in the present study based on our inclusion criteria. [file 2433-3298-4-4-0387-s001.pdf]

# Supplementary fig 2.

A. 1POD

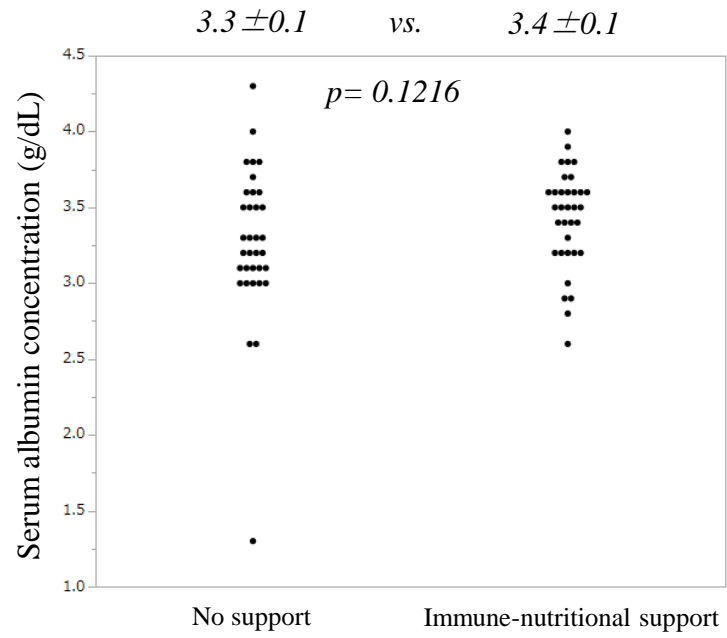

B. 3POD

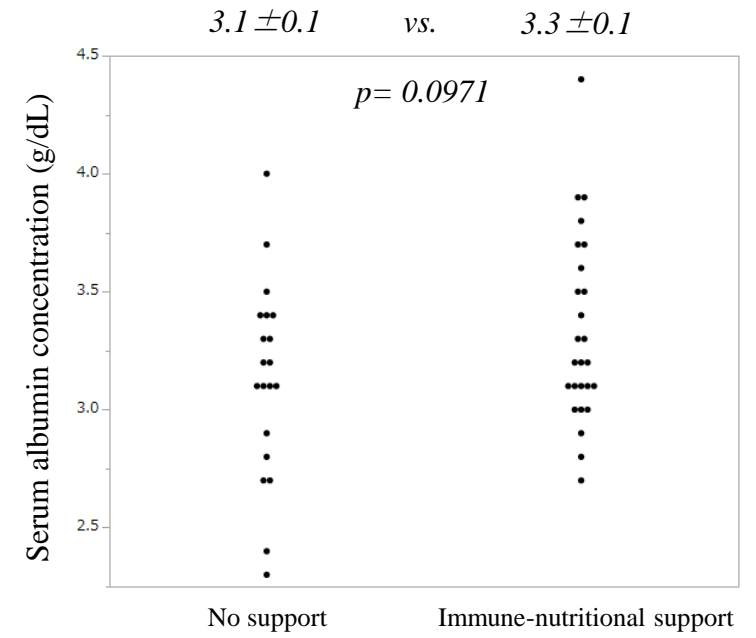

C. 1POD

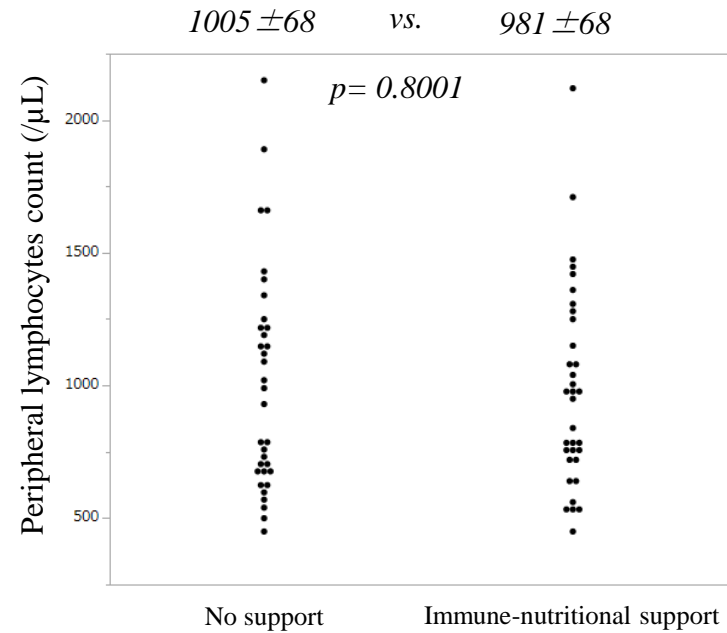

D. 3POD

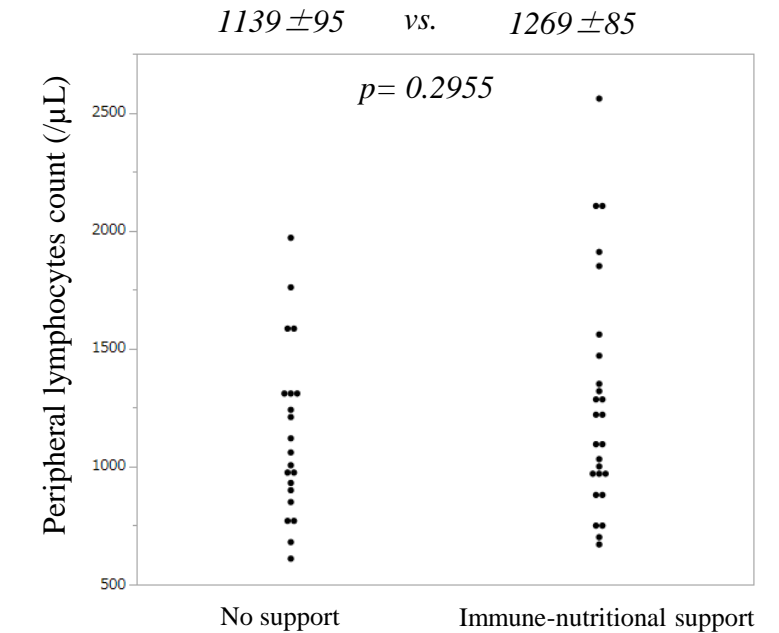

Supplement: Supplementary Figure 2 — The postoperative time course of immune-nutritional parameters including serum albumin concentration on postoperative day (POD) 1 (A) and POD 3 (B) and peripheral lymphocytes count on POD 1 (C) and POD 3 (D) divided by no-support (control) group and preoperative immunonutritional support group. Data are presented as mean ± standard error. [file 2433-3298-4-4-0387-s002.pdf]

Supplementary fig 3.

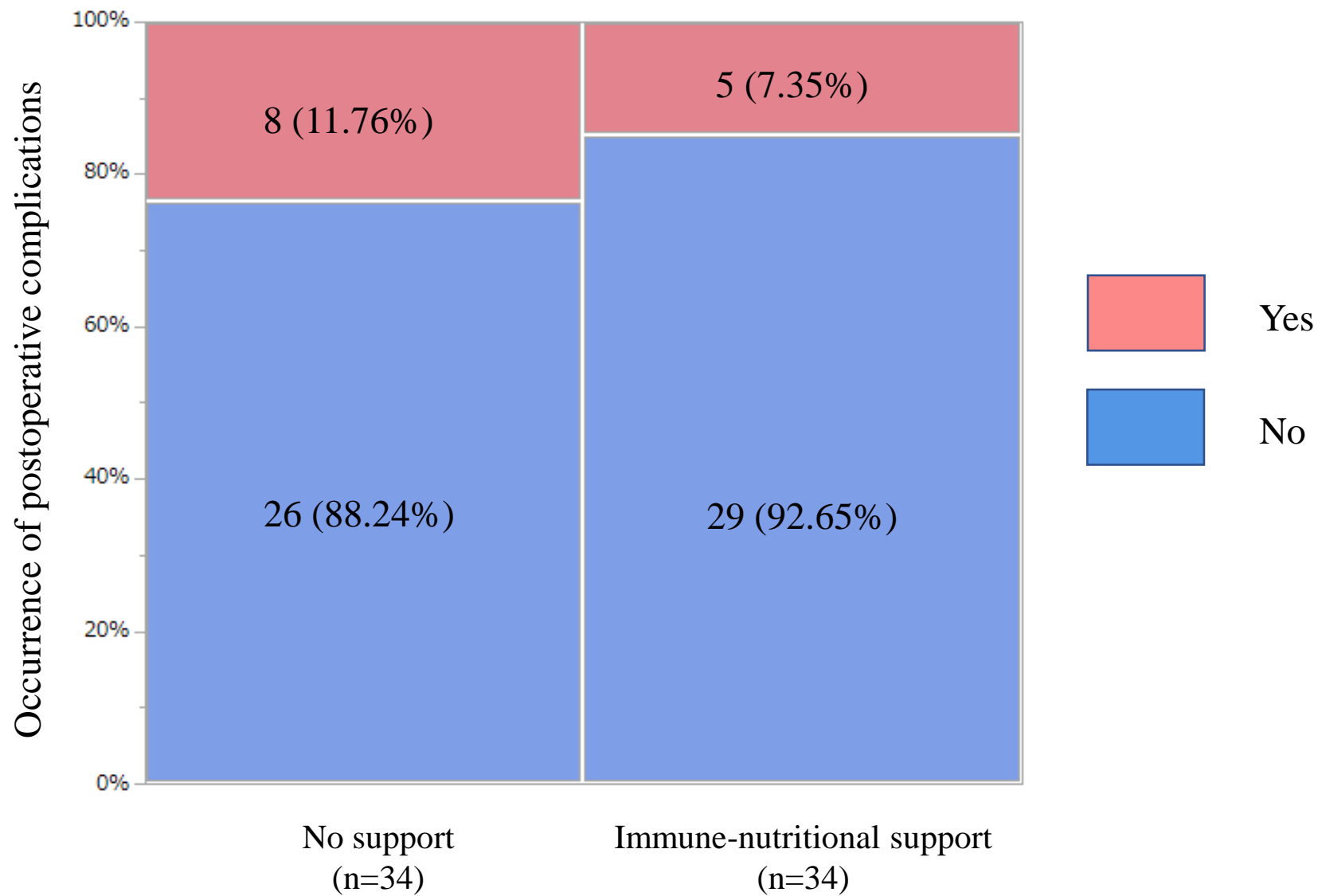

Supplement: Supplementary Figure 3 — A bar chart showing the occurrence of postoperative complications divided by the no-support (control) group and the preoperative immunonutritional support group. [file 2433-3298-4-4-0387-s003.pdf]
